# Acetic Acid-Modulated Room Temperature Synthesis of MIL-100 (Fe) Nanoparticles for Drug Delivery Applications

**DOI:** 10.3390/ijms24021757

**Published:** 2023-01-16

**Authors:** Mengli Ding, Jingwen Qiu, Stéphan Rouzière, Christophe Rihouey, Luc Picton, Ruxandra Gref

**Affiliations:** 1 Institut des Sciences Moléculaires d’Orsay, Centre National de la Recherche Scientifique (CNRS), Université Paris-Saclay, 91405 Orsay, France; 2 Laboratoire de Physique des Solides, CNRS, Université Paris-Saclay, 91405 Orsay, France; 3 Université Rouen Normandie, INSA Rouen Normandie, CNRS, Polymères, Biopolymères, Surfaces (PBS) UMR 6270, 76000 Rouen, France

**Keywords:** room temperature synthesis, metal organic frameworks (MOFs) nanoparticles, acetic acid modulator, cyclodextrin polymers, drug delivery system

## Abstract

Due to their flexible composition, large surface areas, versatile surface properties, and degradability, nanoscale metal organic frameworks (nano MOFs) are drawing significant attention in nanomedicine. In particular, iron trimesate MIL-100 (Fe) is studied extensively in the drug delivery field. Nanosized MIL-100 (Fe) are obtained mostly by microwave-assisted synthesis. Simpler, room-temperature (RT) synthesis methods attract growing interest and have scale-up potential. However, the preparation of RT MIL100 is still very challenging because of the high tendency of the nanoparticles to aggregate during their synthesis, purification and storage. To address this issue, we prepared RT MIL100 using acetic acid as a modulator and used non-toxic cyclodextrin-based coatings to ensure stability upon storage. Hydrodynamic diameters less than 100 nm were obtained after RT synthesis, however, ultrasonication was needed to disaggregate the nanoparticles after their purification by centrifugation. The model drug adenosine monophosphate (AMP) was successfully encapsulated in RT MIL100 obtained using acetic acid as a modulator. The coated RT MIL100 has CD-exhibited degradability, good colloidal stability, low cytotoxicity, as well as high drug payload efficiency. Further studies will focus on applications in the field of cancer therapy.

## 1. Introduction

Since their discovery in 1989, metal organic frameworks (MOFs), built from organic linkers and metal ions, have attracted growing interest for a variety of applications, including catalysis, gas storage, energy, and more recently, biomedicine [1,2,3,4,5,6]. Nanoscale MOFs (nanoMOFs) were employed in the drug delivery field for the treatment of cancer, infections, diabetes, wounds, and other severe diseases [7,8,9,10]. NanoMOFs have advantageous features for drug loading, such as versatile compositions and topologies, and large surface areas. In particular, biocompatible and biodegradable MIL-100(Fe) (MIL stands for Material of Institute Lavoisier) are among the most studied nanoMOFs and have been well-documented in vivo toxicity and biocompatibility data [11]. MIL-100(Fe) with the general formula Fe(III)_3_ O(H_2_O)_2_F · {C_6_H_3_(CO_2_)_3_}_2_ · *n*H_2_O (*n* ~ 14.5) is assembled from trimesate organic linkers and iron trimers, forming an open porous structure with cages of around 5 and 9 Å [12].

The first synthesis of MIL-100(Fe) crystals was carried out at 150 °C for 6 days employing two corrosive acids, HF and HNO_3_ [12]. The purification lasted 3 h at 80 °C. The first nanoscale MIL-100(Fe) for biomedical application was reported in 2009 [13]. They were prepared using HF and needed extensive purification. Lately, to achieve a sustainable synthesis, HF-free MIL-100(Fe) was prepared using a microwave-assisted hydrothermal method [14]. At 130 °C, the reaction lasted for only 6 min in pressurized reactors. However, this method requires specialized equipment.

More recently, research was oriented to the room temperature synthesis of iron-based MIL-100(Fe) (RT MIL100) using common laboratory vessels [15,16,17,18,19]. The few examples in the literature demonstrate that it is possible to obtain RT MIL100 by a simple and scalable “green” (organic solvent-free) method.

The first reported RT MIL100 was prepared in a basic environment in 2017 by simply mixing a solution of trimesic acid and an aqueous solution of iron(II) chloride. The resulting RT MIL 100 was further used for photocatalysis [15]. In 2020, Devi et al. optimized the synthesis by dissolving trimesic acid in sodium hydroxide solutions with different molar concentrations [20]. They reported the application of RT MIL100 as a drug carrier to load and release furosemide. RT MIL100 was nontoxic to normal cells at low concentrations, but their cytotoxicity increased with the dose and the incubation time. Furthermore, Barjasteh et al. used poly(ethylene glycol) (PEG)-coated RT MIL 100 of around 240 nm to incorporate dacarbazine and studied their anti-cancer potential against human melanoma cells [21]. Bimetallic RT MIL 100 was recently synthesized by Steenhaut et al. [18]. The incorporation of Cu(I) drastically lowered the thermal stability of the resulting MOFs, while the incorporation of V, Al, and Ti induced the formation of mesopores.

In 2021, Panchal et al. reported a low-temperature preparation strategy for Fe-, Al- and Ti-based MOFs under mild conditions [22]. RT MIL 100 was synthesized by a “green” method by simply adding iron (III) nitrate nonahydrate into trimesic acid aqueous solutions, opening up possibilities for scaling up. The reported average hydrodynamic diameters were 242 ± 72 nm, and the polydispersity index was 0.201. In contrast, scanning electron microscope (SEM) micrographs showed particles with an average size of 84 ± 13 nm which were probably aggregated.

In conclusion, several challenges remain to be addressed in RT MIL 100 synthesis for drug delivery applications: (i) obtaining hydrodynamic diameters of less than 200 nm is suitable for intravenous administration; (ii) nanoparticles should be non-toxic and (iii) stable upon storage and in biological media.

To date, several reports demonstrate the interest of modulators, such as acetic acid, benzoic acid, formic acid, and dichloroacetic acid, which, added in the MOF’s reaction mixture, allowed for control over their size, shape, and polydispersity [23,24,25,26]. In 2011, Schaate et al. used acetic acid and benzoic acid as modulators to regulate the size and morphology of Zr-based MOFs by controlling the nucleation rate [24]. In 2017, Morris et al. used modulators to control the colloidal stability and polydispersity of UiO-66 MOFs [23]. Acidic modulators with low pKa employed in high concentrations competed with the organic linkers and eventually replaced them in the MOF 3D structures. These generated defects within the UiO-66 structure, which improved the colloidal stability. However, to the best of our knowledge, the acid-modulated synthesis of RT MIL 100 with controllable sizes has not been studied yet.

Nanosized MIL-100(Fe) is widely reported to be unstable [27,28]. Surface modification strategies were developed to prevent MOFs from aggregation, including coating with lipids [28,29,30], polymers [31,32,33], and cyclodextrins (CDs) [27,34,35]. A particular focus was on nanoMOFs’ surface engineering with CD derivatives [27,34,35,36]. CDs are water-soluble cyclic oligosaccharides named α-, β-, and γ-CD, according to the number of their constitutive glucose units (6, 7, or 8, respectively) [37]. NanoMOFs coated with CD-based polymers not only conferred good stability upon storage, but also allowed drug incorporation within the coatings based on the formation of inclusion complexes between CDs and drugs, such as doxorubicin.

Notably, in 2015, Gref and collaborators developed nanoscale MIL-100(Fe) coated with phosphate-CDs (CD-P) [35]. The bulky CD-P molecules could not penetrate through the hexagonal windows of the MOFs, but were firmly anchored to their surface through cooperative coordination with available iron sites at the external surface. The presence of the coating did not interfere with the nanoMOF drug-loading capacity. Based on this approach, the team further synthesized biodegradable CD-based oligomers in an attempt to obtain thicker and more functional coatings. To do so, γ-CD was crosslinked with citric acid by a green strategy devoid of organic solvents [27]. The resulting CD-citrate oligomers allowed enhanced interactions with the MOF cores as compared to their monomeric form (CD). They could also be easily grafted with functional moieties such as PEG, and fluorescent molecules. However, the highly acidic character of the citrate-based CD oligomers is considered a drawback for biomedical applications, and other crosslinking agents such as malic acid were proposed as alternatives [38].

Thus, our aim here was to develop RT MIL100 with controllable sizes, good stability, and low toxicity for drug delivery applications. We propose the preparation of RT MIL100 using a simple, green, and sustainable method using acetic acid as a modulator. By tuning the molar ratio (R) of the modulator to a trimesic acid, the reaction time, and the reaction speed, the hydrodynamic diameters of RT MIL 100 could be varied in the range of 40–200 nm. Best results were obtained R = 53, and the resulting RT MIL 100 were further coated with oligomers made of β-CD cross-linked with malic acid (CD-MO). These new oligomers are strongly associated with RT MIL100 by virtue of the strong affinity between the iron sites at the external RT MIL100 surface and the carboxyl groups on CD-MO. CD-MO-coated RT MIL100 (CD-MO@RT MIL100) exhibited similar crystallinity and drug-loading abilities as the uncoated ones. Drug encapsulation and release studies were carried out with adenosine 5′-monophosphate disodium salt (AMP). The AMP encapsulation efficiency within RT MIL100 and CD-MO@RT MIL100 reached 99% and 98%, respectively. The drug was released following RT MIL100 degradation. Both synthesized RT MIL100 and CD-MO showed low toxicity in macrophage J744 cells. This study opens new perspectives for the preparation of size-controllable RT MIL100 with good stability as well as low toxicity, with possible potential applications in biomedical and drug delivery systems.

## 2. Results and Discussion

**Figure 1:**
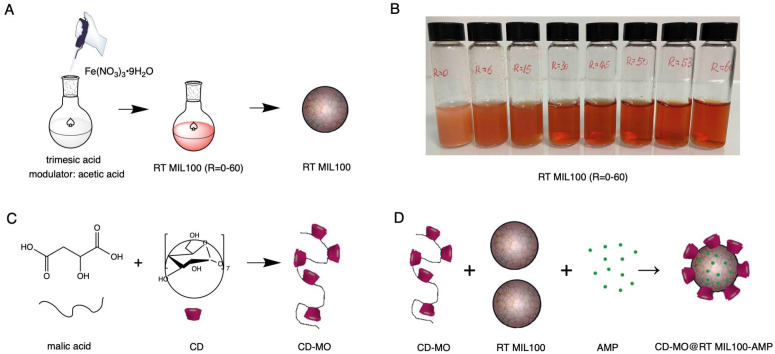
Schematic representation of the synthesis of (**A**) RT MIL100 using acetic acid as a modulator, trimesic acid as an organic linker, and iron (III) nitrate; (**B**) photos of the reaction mixtures immediately after mixing the reactants. Effect of the amount of modulator on the color and turbidity of the reaction mixture. The color variation of modulated RT MIL100 (R = 0–60); (**C**) CD-MO oligomers obtained by crosslinking CD with malic acid; (**D**) core-shell and drug-loaded nanoparticles (CD-MO@RT MIL100-AMP) obtained by simply mixing the components of the system at room temperature and in water.

First, we present the synthesis of RT MIL 100 (Figure 1A,B) and CD-MO (Figure 1C), and then we describe the coating method (Figure 1D).

### 2.1. Preparation of RT MIL100: Effect of the Acetic Acid Modulator

#### 2.1.1. Synthesis of RT MIL100 without Modulators

In the first step, RT MIL100 was prepared by adapting a previously reported method and without using a modulator [22]. The nanoparticles, named RT MIL100 (R = 0), were synthesized in mild conditions by adding a solution of iron (III) nitrate into a solution of trimesic acid at room temperature (Figure 1A). Time zero corresponds to the mixing of the two solutions. The reaction started instantly and the solution became immediately turbid, as shown in Figure 1B (R = 0). Samples of these mixtures were withdrawn and diluted with water to analyze the nanoparticle size distribution directly by DLS and TEM.

**Figure 2:**
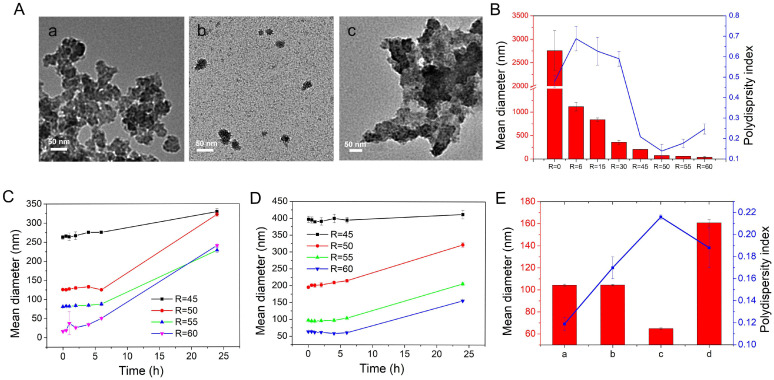
(**A**) TEM images of (**a**) RT MIL100 (R = 0) without purification, (**b**) RT MIL100 (R = 53) without purification, (**c**) RT MIL100 (R = 53) synthesized at 500 rpm, during 4 h and after purification by centrifugation; (**B**) mean diameters and PDI of RT MIL100 (non-sonicated) synthesized at different ratios of acetic acid and trimesic acid (R = 0~60); (**C**) mean diameters of RT MIL100 (non-sonicated) synthesized at 500 rpm at the ratios of R = 45, 50, 55, 60; (**D**) mean diameters of RT MIL100 (non-sonicated) synthesized at 300 rpm with the ratios of R = 45, 50, 55, 60; (**E**) mean diameters and PDI of RT MIL100 (R = 53) prepared at different conditions ((**a**): samples at 0 h without purification, (**b**): samples at 4 h without purification, (**c**): the supernatant of the samples at 4 h after centrifugation, (**d**): samples at 4 h after purification and ultrasonication).

Figure 2A(a) shows that RT MIL100 typically exhibits a tiny sphere-like structure with an average size of around 40–50 nm, as calculated using the Image J software. However, the tiny nanoparticles were strongly aggregated and impossible to disaggregate by diluting in water. In agreement with this observation, DLS investigations showed large hydrodynamic diameters of >2500 nm and PDI > 0.5, as illustrated in Figure 2B (R = 0). Additionally, the suspensions sedimented readily (Figure S1).

Possibly, this aggregation may be attributed to the very quick contact and reaction between the reactants, making it hard to generate individual nanoparticles. Notably, after applying an intense and long-term sonication process (at least 1 h at 25 °C), the hydrodynamic diameters of RT MIL100 (R = 0) could be decreased to 200–300 nm, which was consistent with the reported studies [22]. However, ultrasonication is not only energy- and time-consuming, but also inefficient in producing nanoparticles with low PDI.

Therefore, our aim here was to set up a robust method to control the reaction process and reduce the size of RT MIL100 from the very beginning of the synthesis reaction.

#### 2.1.2. Synthesis of Modulated RT MIL100

Modulators were employed in MOF synthesis to control the reaction speed. Among the variously reported modulators, acetic acid was chosen here as it is less toxic. The concentration of acetic acid was the first parameter investigated to regulate the synthesis process, and various molar ratios R of acetic acid to organic linkers trimesic acid were investigated (R = 0–60). The modulator was mixed with the organic linker and then reacted with iron (III) nitrate.

Figure 1B shows the color variation of modulated RT MIL100 immediately after starting the reaction. All modulated RT MIL100 suspensions present a red-orange color and a pH of 2~3. The turbidity of the RT MIL100 suspensions decreased with the increase of the ratio R, indicating a reduction in particle aggregation. In line with the observations, sedimentation studies (Figure S1) showed that modulated RT MIL 100 were more stable than the non-modulated ones. After storage at room temperature for 2 days, sedimentation of RT MIL100 (R = 6) was significantly reduced as compared to RT MIL100 (R = 0). Notably, the RT MIL100 (R = 15–60) suspensions were stable during this time with no observable sedimentation.

Similarly, as in the case of RT MIL100 (0 h), samples of the reaction mixture were taken and diluted to analyze their size distribution. The mean diameters of modulated RT MIL100 were reduced significantly by employing the modulator (Figure 2B). At ratios of 6–30, the mean diameters of modulated RT MIL100 decreased from 1116 nm to 357 nm, suggesting a lower aggregation. Moreover, the hydrodynamic diameters of RT MIL100 decreased from 204 nm to 38 nm when R increased from 45 to 60. These data are in agreement with the aspect of the nanoparticles’ suspensions (Figure 1B), showing that the turbidity of the suspensions was drastically reduced when increasing R, which is illustrative of a reduced aggregation. Modulators compete with organic linkers to coordinate with iron sites, thus slowing the reaction speed, which in turn allows for better control of the reaction process, making it possible to reduce the nanoparticles’ aggregation to produce individual RT MIL100.

The smallest hydrodynamic diameters (<200 nm) and lowest PDI values (<0.3) (Figure 2B) were obtained with the ratios of 45–60, which were further selected to investigate another critical parameter that affects the modulated process, the reaction time. Samples were taken from the reaction mixtures at different times (0–24 h), diluted and analyzed directly by DLS. Figure 2C shows that the mean diameters of RT MIL100 remained practically unchanged in the first 6 h, but then significantly increased with time up to 24 h. For example, at the ratio of R = 50, the mean diameters of RT MIL100 were kept ~125 nm within 6 h, and then reached ~320 nm at 24 h. Likewise, similar profiles were obtained when the stirring speed was decreased from 500 rpm to 300 rpm and for the same ratios of 45–60 (Figure 2D). As expected, the mean hydrodynamic diameters of RT MIL100 prepared at 300 rpm were much larger than those prepared at 500 rpm, indicating the hydrodynamic parameters play a role in avoiding and/or dispersing the aggregates.

PDI is an essential parameter characterizing the nanoparticles’ size distribution. Figure S2 displays the PDI of RT MIL100 synthesized at 500 rpm and 300 rpm. In both cases, the PDI values increase with the reaction time. For instance, at the ratio R = 50, the PDI of RT MIL100 synthesized at 500 rpm increased from 0.14 (0 h) to 0.26 (24 h). Likewise, for RT MIL100 synthesized at 300 rpm, the PDI increased from 0.18 (0 h) to 0.27 (24 h). We hypothesize that the longer the reaction time, the higher the probability that the nanoparticle aggregate, resulting in higher PDI values.

In conclusion, optimization studies allowed for a conclusion that the best results (DLS size of ~100 nm and the lowest PDI of 0.12) with the modulated RT MIL100 synthesized were obtained with the high stirring speed at 500 rpm at a ratio of R = 53 and with a reaction time of 4 h. They were selected for further study.

#### 2.1.3. Purification of Modulated RT MIL100

Purification is an essential step in MOF synthesis, and successive centrifugation is a commonly used approach to remove unreacted residues. Firstly, we attempted to purify RT MIL100 by centrifugation. It was easy to collect and wash the RT MIL100 (R = 0) synthesized without modulators by centrifugation in mild conditions (8000× *g*, 5 min). However, the modulated RT MIL100 (R = 30–60) was smaller and harder to be collected by sedimentation in the same condition (8000× *g*, 5 min). For each R, both the centrifugation speed and time needed to be optimized. For example, RT MIL100 (R = 53) was collected at 15,000× *g*, 1 h, in agreement with data reported for other MOFs [23]. Notably, after a first centrifugation step to remove precipitates (15,000× *g*, 1 h), the nanoparticles were repeatedly washed at 15,000× *g*, 10 min with water and ethanol, and the pH of the suspensions was fixed to 4~5.

The hydrodynamic diameters and PDI of purified RT MIL100 (R = 53) were analyzed from the beginning of the reaction to the end. As illustrated in Figure 2E, the mean diameters were around 104 nm after 4 h. TEM images of RT MIL100 (R = 53) (Figure 2A(b)) further confirmed that RT MIL100 (R = 53) was well-dispersed, with sizes of around 56 nm as calculated by Image J. However, after washing by centrifugation, the nanoparticles ineluctably aggregated because of the strong centrifugal forces and their intrinsic tendency to stick together (Figure 2A(c)). Ultrasonication for 30 min was indispensable to disperse the purified RT MIL100 nanoparticles. After this, the mean hydrodynamic diameters measured by DLS were 160 nm. Additionally, the supernatant of RT MIL100 generated in the first centrifugation step without ultrasonics was analyzed, and the mean diameters and PDI were 65 nm and 0.2, respectively (Figure 2E(c)). Therefore, we concluded that the smallest RT MIL100 was removed by purification, resulting in larger and more homogeneous RT MIL100.

In a nutshell, we found that: (1) the size of RT MIL100 (R = 0) was smaller than that of MIL100 (Fe) nanoMOFs produced by microwave-assisted synthesis, but they had a high tendency to aggregate; (2) Modulator allowed to dramatically reduce the aggregation process at the beginning. However, aggregation still occurred at long reaction times; (3) Aggregation could be reduced by increasing the shearing rate; (4) However, the purification process was very delicate and a final sonication was necessary to produce nanoparticles with low PDI and sizes around 160 nm.

### 2.2. Characterization of RT MIL100

**Figure 3:**
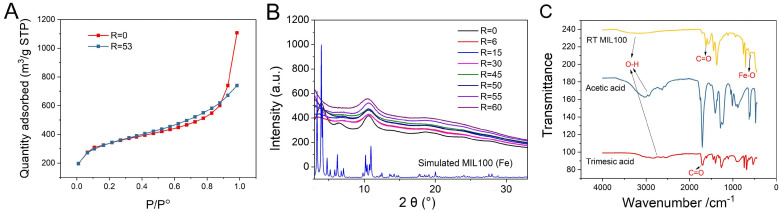
(**A**) Porosimetry characterization of RT MIL100 (R = 0) and RT MIL100 (R = 53) at 77.3 K; (**B**) PXRD patterns of RT MIL100 synthesized at different ratios of the modulator and trimesic acid (R = 0–60); (**C**) ATR IR spectrums of RT MIL100 (R = 53), acetic acid, and trimesic acid.

A series of investigations were carried out to characterize the purified RT MIL100. Firstly, the porosity of RT MIL100 was evidenced using N_2_ adsorption-desorption experiments, and the BET surface areas of RT MIL100 (R = 0) and RT MIL100 (R = 53) were calculated (Figure 3A). The BET surface area of RT MIL100 (R = 0) was 1218 m^2^/g, while the BET surface area of modulated RT MIL100 (R = 53) was 1198 m^2^/g, and there is no significant variation (<10%) between them. These data suggest that the surface areas of RT MIL100 were not affected by the modulator.

Powder X-ray diffraction (PXRD) studies of RT MIL100 synthesized at the ratios of R = 0–60 were carried out. Figure 3B illustrates that the crystalline structures of RT MIL100 (R = 0) were consistent with the reported one [22]. The crystalline structures of acetic acid-modulated RT MIL100 (R = 6~60) were homologous, and they exhibited fewer crystalline features than RT MIL100 without a modulator. This may be caused by the competition between modulators and organic linkers in the synthesis process, thus generating more defects within MOFs’ structure, thus affecting the crystallization of RT MIL100.

ATR infrared analysis allowed further investigation of the chemical structures and compositions of RT MIL100. Figure 3C shows an evident shift of the C=O bond absorption peak from 1761 cm^−1^ (trimesic acid) to 1621 cm^−1^ (RT MIL100), corresponding to the coordination of trimesic acid with Fe (III), thus indicating the successful synthesis of RT MIL100, in agreement with reported data in the case of microwave-MIL-100(Fe) [39,40]. Stretching vibrations of O-H bonds from trimesic acid (2850 cm^−1^) and acetic acid (3040 cm^−1^) were shifted to around 3200 cm^−1^ (RT MIL100), signifying the presence of hydrogen bonding after coordination with Fe^3+^. The stretching vibration of the Fe−O bond is at 613 cm^−1^ [39]. Notably, the low-intensity peaks of trimesic acid and acetic acid within RT MIL100 show the effective removal of these reactants from the purified nanoparticles.

In addition, the IR spectra of the purified RT MIL100 (R = 0–60) were consistent, indicating that the modulators did not alter the compositions of RT MIL100 (Figure S3).

### 2.3. Synthesis of CD-MO

CD-MO was synthesized based on our previous synthesis strategy of CD-citrate oligomers with minor modifications [27]. Briefly, β-CD and malic acid were mixed and solubilized in an aqueous solution, which was further concentrated by evaporating water under reduced pressure. Then, the reaction was carried on at 160 °C, 170 °C, or 180 °C. Table 1 summarizes the characteristics of the obtained CD-MO samples, after their purification by dialysis.

At 160 °C (entries 1–5), the yield of CD-MO increased from 24% to 38% with the increase of the reaction time. Likewise, with the same heating time of 25 min (entries 1, 7, 11), the yield of oligomers increased from 24% to 32% and 35%, as the temperature increased from 160 °C to 170 °C and 180 °C. Notably, at the temperature of 170 °C, the yield of CD-MO (30–33%) did not change significantly with the increase of the heating time (entries 6–9). The yield of oligomers decreased to 24 % (entry 12), with a heating time of 30 min at 180 °C. This is due to the higher amounts of insoluble fractions generated in the synthesis process, as reported in our previous research [27].

The dispersity of molar masses (Ð) is an essential parameter to assess the quality of the obtained CD-MO. The polydispersity of CD-MO increased from 2.2 to 3.9 with the increase of the heating time at 160 °C (entries 1–5), Table 1. Whatever the reaction time, Ð were higher than 3.0 at 170 and 180 °C.

**Figure 4:**
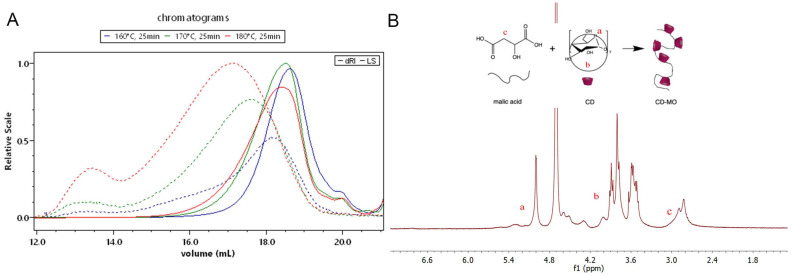
(**A**) SEC/MALS/DRI chromatograms of CD-MO synthesized at 160 °C (purple), 170 °C (green), and 180 °C (red) for 25 min showing both dRI and LS signals; (**B**) ^1^H NMR spectrum of CD-MO synthesized at 160 °C, 25 min. Based on the integrated signals of CD units and malic acid, the evaluated ratio of CD and malic acid moieties was 1:3.

Figure 4A shows typical SEC/MALS/DRI chromatograms of CD-MO obtained with a reaction time of 25 min at 160 °C, 170 °C, and 180 °C. The retention times at the maximum of dRI peak were shifted from 18.6 to 18.4 when increasing the temperature, reflecting an increase in the molar mass. In addition, information from the light-scattering detector (LS, dotted line) shows a population in CD-MO synthesized at 170 °C and mainly at 180 °C eluted at 13.5 mL, with high hydrodynamic volume (i.e., high molar masses) which is not detected by the differential refractive index (dRI) detector. This indicates that this population is in the minority. However, its presence compromises the Ð. In conclusion, the selected reaction conditions were 160 °C, 25 min, resulting in a CD-MO with the lowest PDI (2.2) and M_w_ around 12,000 g/mole.

^1^H-Nuclear magnetic resonance (NMR) was used to analyze the CD-MO chemical composition of all CD-MO oligomers. A typical example is shown in Figure 4B in the case of optimal CD-MO with PDI 2.2. CD-MO displays signals arising from both its β-CD and malic acid components. In particular, signal a (δ 5.9–4.9 ppm) represents 7 anomeric protons of CD units, and signal c (δ 3.4–2.7 ppm) corresponds to the 2 protons of the malic acid. The molar ratio of malic acid and CD in CD-MO was calculated by integrating the peaks assigned to signals a and c. Signal b (δ 4.9–3.3 ppm) corresponds to CD units. The calculated molar ratio CD: malic acid was 1:3 (Table 1), corresponding to a global composition of (CD)4-(malic)12.

### 2.4. Physicochemical Characterizations of CD-MO@RT MIL100

**Figure 5:**
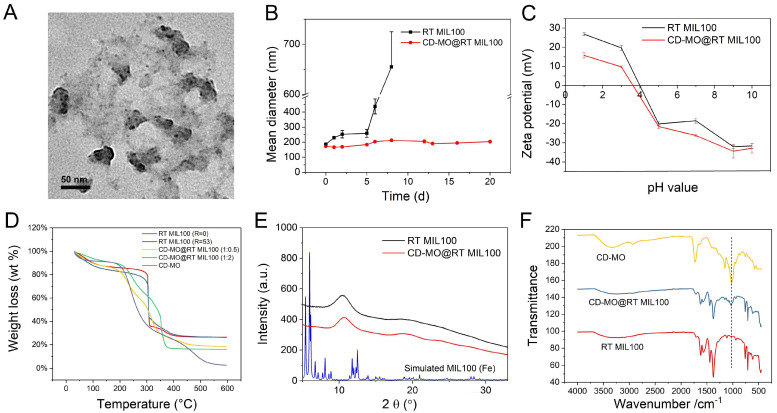
(**A**) TEM image of CD-MO@RT MIL100 (R = 53); (**B**) Stability of RT MIL100 and CD-MO@RT MIL100 in three weeks. Samples were stored at 4 °C in water, and the hydrodynamic diameters were measured by DLS; (**C**) Zeta potentials of RT MIL100 and CD-MO@RT MIL100 at pH 1–10; (**D**) TGA analysis of RT MIL100 (R = 0 and R = 53), CD-MO, and CD-MO@RT MIL100. The weight ratios of CD-MO: RT MIL100 used for coating were 1:0.5 and 1:2; (**E**) PXRD pattern of RT MIL100 (R = 53) coated or not with CD-MO; (**F**) ATR IR spectrum of RT MIL100, CD-MO, and CD-MO@RT MIL100.

CD-MO were spontaneously coated on RT MIL100 at room temperature with a “green” organic-free method by simply incubating them in aqueous solutions overnight. A set of complementary approaches were employed for the physicochemical characterizations of CD-MO@RT MIL100. First, DLS was used to analyze the hydrodynamic diameters of RT MIL100 after coating with CD-MO. Compared with the naked RT MIL100 (161 nm), the mean diameters of CD-MO@RT MIL100 was 173 nm, showing that the CD-MO coating had a reduced effect on the size of RT MIL100 (size variation < 10%). TEM images of CD-MO@RT MIL100 (Figure 5A) also showed similar morphologies and sizes.

Additionally, the BET surface area of CD-MO coated RT MIL100 was 1144 m^2^/g (Figure S4), without noticeable difference with the naked RT MIL100 (1198 m^2^/g). This indicated that the CD-MO coating did not affect the porosity of RT MIL100.

Colloidal stability is crucial for the storage and applications of nanoMOFs. RT MIL100 and CD-MO@RT MIL100 aqueous solutions were stored at 4 °C, and the hydrodynamic diameters were monitored by DLS over 3 weeks. As shown in Figure 5B, the naked RT MIL100 aggregated within one week only, when their size became larger than 600 nm. In contrast, the CD-MO coated RT MIL100 was stable within three weeks without significant size variation in aqueous media. This suggested that the oligomer coating could efficiently avoid RT MIL100’s aggregation.

The surface charge of RT MIL100 before and after coating with CD-MO was investigated. Zeta potential (ZP) was measured in a pH range of 1 to 10 (Figure 5C). The ZP of RT MIL100 evolved from + 27 mV to −32 mV in this pH range. After coating with CD-MO, the ZP of CD-MO@RT MIL100 varied from +16 mV to −33 mV with the same pH range. These values are in line with previously reported values [27]. In addition, as shown in Figure S5, the ZP of CD-MO coated RT MIL100 was much lower (−21 mV) as compared with the ZP of RT MIL100 aqueous suspensions (−12 mV). This indicates a better electrostatic stabilization of the coated RT MIL100, favorable for their colloidal stability.

The CD-MO coated RT MIL100 was further investigated by TGA (Figure 5D). Three weight loss steps were found within the 30–600 °C temperature range. The weight loss before 200 °C was caused by the evaporation of free and associated water. From 200 °C, CD-MO started to degrade. The degradation of RT MIL100 occurred in the range of 300–500 °C. There were no apparent differences in the thermograms of RT MIL100 (R = 53) and RT MIL100 (R = 0). Notably, CD-MO degraded completely in the heating process. The calculated associated amounts of CD-MO were 28 wt % (1:0.5) and 43 wt % (1:2). This shows the strong coordination between CD-MO and iron sites on RT MIL100.

PXRD investigations enabled analyzing the crystalline structure of RT MIL100 before and after coating with oligomers. As shown in Figure 5E, the PXRD patterns of RT MIL100 and CD-MO@RT MIL100 were consistent, with no visible difference. This suggests that the coating layer of CD-MO does not affect the crystalline structure of RT MIL100.

The IR spectrums of RT MIL100 coated or not with CD-MO were further analyzed. As shown in Figure 5F, CD-MO displayed a characteristic peak at 1020 cm^−1^ (primary alcohol), while no peak was observed for RT MIL100 in this region. The distinct CD-MO peak was found in the CD-MO@RT MIL100, demonstrating the successful coating with the oligomers.

### 2.5. Drug Loading, Release, and RT MIL100 Degradation Study

The phosphate drug adenosine monophosphate (AMP) was chosen to investigate drug encapsulation, release, and related RT MIL100 degradation. AMP was loaded by simply incubating with RT MIL100 and CD-MO@RT MIL100, at a weight ratio of 1:5. The amount of non-encapsulated drug was quantified by HPLC in the supernatants recovered after impregnation. The encapsulation efficiencies in RT MIL100@AMP and CD-MO@RT MIL100-AMP were 99% and 98%, respectively (Figure S6). The payload efficiency of RT MIL100@AMP and CD-MO@RT MIL100-AMP reached 19.7% and 19.6%, respectively.

This efficient drug incorporation is in line with reported data in the case of drug entrapment in MIL-100(Fe) nanoMOFs prepared by microwave-assisted synthesis [41,42]. It shows that a phosphate drug readily coordinates with the iron sites in the RT MIL100 framework. These results also indicated that the CD-MO coating did not affect the AMP loading.

**Figure 6:**
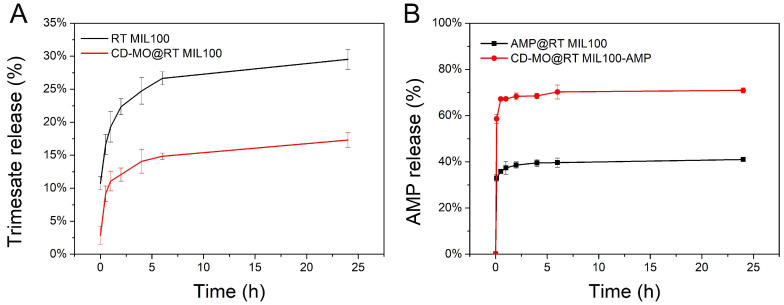
(**A**) Trimesate release from RT MIL100 and CD-MO@RT MIL100 in 1 mM PBS at 37 °C; (**B**) AMP release from AMP@RT MIL100 and CD-MO@RT MIL100-AMP in 1 mM PBS at 37 °C.

Drug release and RT MIL100 degradation were studied in parallel during incubation at 37 °C in 1 mM PBS (Figure 6). RT MIL100 degradation was evaluated based on the release of its constitutive ligand, trimesate. It is known that free phosphates in phosphate buffer saline (PBS) solution compete with organic linkers to coordinate with the unsaturated Lewis acid sites (CUS) of MIL-100(Fe), leading to degradation and drug release [28,43]. This was also the case with RT MIL100 (Figure 6A). Moreover, the trimesate release from RT MIL100 was significantly higher than from CD-MO@RT MIL100. For example, 25% of the total trimesate amount in RT MIL100 was released within 4 h and finally reached 30 % after 24 h. In contrast, only 14% of trimesate was released from CD-MO@RT MIL100 within 4 h and reached 17% at 24 h. We hypothesized that the CD-MO coating might hinder the accessibility of the CUS to the phosphates, thus reducing the RT MIL100 degradation. We further studied the AMP release profiles from RT MIL100 and CD-MO@RT MIL100. As shown in Figure 6B, AMP was rapidly released from both coated and uncoated nanoparticles. The incomplete drug release (plateaus at around 40 and 70%) could be due to insufficient competition with phosphates in PBS, as reported previously [44].

### 2.6. Toxicity Study

The toxicity of newly synthesized RT MIL100 and CD-MO was evaluated with the macrophage J744 cell line. We determined the half-maximal inhibitory concentration (IC50), which is the concentration of a material needed to inhibit a biological process by 50 %, as an indicator of the materials’ toxicity.

**Figure 7:**
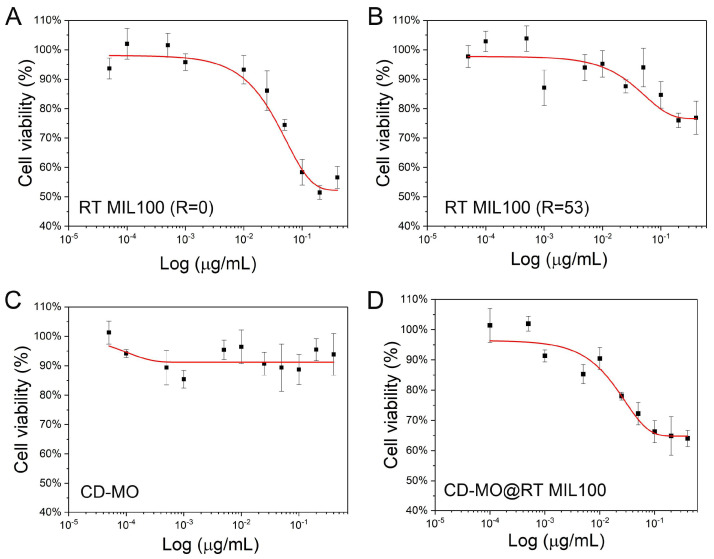
Macrophage J744 cells dose-response curves after treatment with nanoparticles (MTT assay). Cell viability was tested after treatment with (**A**) RT MIL100 (R = 0); (**B**) RT MIL100 (R = 53); (**C**) CD-MO; (**D**) CD-MO@RT MIL100. Results were exploited as mean ± SD for IC50 values obtained from three or four repeats for each individual compound.

Cell toxicity of RT MIL100 without modulators was first determined. As shown in Figure 7A, 75% of cell viability was obtained with 50 μg/mL RT MIL100 (R = 0) treatment. The IC50 concentration of RT MIL100 (R = 0) on J744 cells was 400 μg/mL, suggesting the low toxicity of RT MIL100 (R = 0). Surprisingly, as demonstrated in Figure 7B, when cells were treated with modulated RT MIL100 (R = 53), 75% of cell viability was obtained with 400 μg/mL RT MIL100 (R = 53) treatment. RT MIL100 (R = 53) did not demonstrate significant cytotoxic effects on J744 cell lines and no IC50 values were obtained. This could be explained based on their different composition, stability, and size.

Likewise, CD-MO also showed low toxicity and no IC50 values were obtained in the studied concentration range. Figure 7C showed 90% cell viability of J744 cells even after treatment with 400 μg/mL CD-MO, which suggested the potential of CD-MO for DDS application. The cytotoxicity of CD-MO coated RT MIL100 (R = 53) is displayed in Figure 7D. Cell viability of J744 macrophages treated with 400 μg/mL CD-MO@RT MIL100 was 65%, which is an average value of their respective components, CD-MO and RT MIL100 (R = 53) ‘s toxicity, and no IC50 value was obtained in the studied concentration range. In conclusion, these studies demonstrated the low-toxicity of modulated RT MIL100, and of their coating material CD-MO, which exhibit their potential for biomedical applications.

## 3. Materials and Methods

### 3.1. Materials

Trimesic acid, DL- malic acid, sodium phosphate dibasic dodecahydrate (NaH_2_PO_4_·12H_2_O), adenosine 5′-monophosphate disodium salt (5′-AMP-Na_2_), Thiazolyl Blue Tetrazolium Bromide (MTT) and absolute ethanol were obtained from Sigma-Aldrich (Saint Quentin Fallavier, France). Iron (III) nitrate nonahydrate was obtained from Thermo Fisher Scientific (Courtaboeuf, France). Acetic acid was from VWR chemicals (Darmstadt, Germany). β-cyclodextrin (CD) was purchased from Roquette (Lestrem, France). Milli-Q water was obtained from an ELGA water system (Lane End, UK). Dulbecco’s Phosphate Buffered Saline (DPBS) (1X), and Dulbecco’s Modified Eagle Medium DMEM (1X) were from Thermo Fisher Scientific (Montigny-le-Bretonneux, France) and were used for drug release and as cell culture media, respectively. Enzyme Express (1X) TrypLE™, without phenol red (Thermo Fisher Scientific, Montigny-le-Bretonneux, France) was used to detach cells. All products for cell culture studies were sterile.

### 3.2. Synthesis and Characterization

#### 3.2.1. Preparation of RT MIL100 with/without Modulators

The synthesis of RT MIL100 without modulators was carried out by adapting a reported protocol [22]. Solution 1 was prepared by dissolving 33 mg (0.159 mmoL) of trimesic acid in 10 mL Milli-Q water. Solution 2 was prepared by dissolving 96 mg (0.237 mmoL) iron (III) nitrate nonahydrate in 1 mL Milli-Q water. Solution 2 was added dropwise into solution 1 at room temperature under stirring (Figure 1A). The mixture was allowed to react under stirring at 500 rpm for 4 h. The obtained nanoparticles were recovered by centrifugation (8000× *g*, 5 min) and washed three times with Milli-Q water and three times with absolute ethanol. The collected samples were re-suspended in ethanol or dried at 60 °C overnight in an oven for TGA, BET, PXRD, and IR studies.

The synthesis of RT MIL100 with acetic acid as a modulator was carried out based on the above method. Solution 1 was prepared by dissolving 33 mg (0.159 mmoL) trimesic acid in 10 mL Milli-Q water followed by adding acetic acid to reach molar ratios R (acetic acid: trimesic acid) comprised between 0 and 60. Solution 2 was prepared by dissolving 96 mg (0.237 mmoL) iron (III) nitrate nonahydrate in 1 mL Milli-Q water. Solution 2 was dropwise added into solution 1 at room temperature under stirring. The reaction mixture was stirred at 500 rpm or 300 rpm for 0–24 h. Nanoparticles were collected by centrifugation at 15,000× *g* (10–90 min) and washed thrice with Milli-Q water, and thrice with absolute ethanol. The collected samples were re-suspended in ethanol or dried for further studies.

#### 3.2.2. Preparation of CD-MO

CD-MO was prepared by adapting our previous synthesis method of CD-citrate oligomers [27]. NaH_2_PO_4_·12H_2_O was used as catalyst to promote the cross-linking reaction between CD and malic acid under reduced pressure, in which the molar ratios of CD and malic acid were 1:6. Firstly, 600 mg (0.475 mmoL) CD, 382 mg (2.8 mmoL) malic acid, and 360 mg (1.0 mmoL) catalyst were mixed and solubilized in 5 mL MilliQ water. The solution was concentrated by evaporation at 160 °C for 15 min. The resulting dried mixture was heated at 160 °C/170 °C/180 °C for different times (20–90 min) under reduced pressure by connecting the system with a suction pump. A yellowish material was obtained at the end of the reaction. A total of 10 mL of MilliQ water was added to solubilize the product. After 5 min of ultrasonication, the crude was filtered to separate the insoluble fraction from the soluble one. Samples were then dialyzed for 48 h (20 kDa cellulosic membrane, Spectrum Laboratories, Rancho Dominguez, CA, USA), and water was changed four times during this process. The solution was finally dried by rotary evaporation and lyophilized to yield a slightly yellow powder.

#### 3.2.3. Preparation of CD-MO@RT MIL100

Based on our previous studies [27], a mass ratio of 1:2 between oligomers and MOFs was chosen for the coating process. To do so, 1 mL of a suspension of RT MIL100 (1 mg/mL) was added into 1 mL solution containing 0.5 mg/mL CD-MO. The mixture was stirred at room temperature overnight to form CD-MO@RT MIL100. The coated nanoparticles were collected (10,000× *g*, 10 min) and washed with Milli-Q water. They were resuspended in 1 mL MilliQ water for further use.

#### 3.2.4. Preparation of AMP@RT MIL100 and CD-MO@RT MIL100-AMP

First, suspensions (1 mg/mL) of RT MIL100 and CD-MO@RT MIL100 were prepared. They were separately added into 1 mL AMP aqueous solution (0.2 mg/mL) and stirred at room temperature for 24 h. Samples were collected (10,000× *g*, 10 min) and washed with Milli-Q water to remove the non-encapsulated AMP molecules. Finally, AMP@RT MIL100 and CD-MO@RT MIL100-AMP were separately re-suspended in 1 mL water for further studies.

#### 3.2.5. Characterization of RT MIL100

Transmission electron microscope (TEM, MET JEOL 1400 (80 kV), Tokyo, Japan) was utilized to characterize the size and morphology of RT MIL100 and CD-MO@RT MIL100. The TEM grids were firstly treated with PELCO easiGlow™ Glow Discharge Cleaning System. The diluted nanoparticle samples were gently dropped onto the grids and allowed to settle for 2 min. After removing the extra water with tissue paper, samples were transferred into the TEM system for analysis.

Dynamic light-scattering (Nano ZS90, Malvern Panalytical, Worcestershire, UK) operating at 90° was used to determine the hydrodynamic diameter and the polydispersity of the nanoparticles. Each sample was tested 3 times. DLS was also employed to study the colloidal stability of RT MIL100 coated or not with CD-MO. RT MIL100 and CD-MO@RT MIL100 were stored at 4 °C fridges, and analyzed at 0 d, 1 d, 2 d, 5 d, 6 d, 8 d, 12 d, 13 d, 16 d, and 20 d.

Zeta potential (ZP) of RT MIL100 and CD-MO@RT MIL100 was determined with a Zetasizer instrument (Nano ZS90, Malvern Panalytical, Worcestershire, UK) in a pH range of 1–10.

Powder X-ray Diffraction (PXRD) measurements of nanoparticles coated or not with CD-MO were carried out on the MORPHEUS platform at the Laboratoire de Physique des Solides (Université Paris-Saclay, Orsay, France), with a home-made diffraction setup installed on a rotating anode generator (model RUH3R, Rigaku Corp., Tokyo, Japan) at the Cu wavelength (λ_CuKα_ = 0.1542 nm) delivered by a multilayer W/Si mirror (Osmic). Powders were filled in cylindric borosilicate capillaries (diameter of 1 mm, WJM-Glass Müller GmbH, Berlin, Germany). Diffraction patterns were collected on a large-area detector MAR345 (marXperts GmBH, Norderstedt, Germany) with 150 µm pixel size placed at a distance of 250 mm from the sample. The experimental resolution is a Gaussian function with full-width at half-maximum equal to 0.013Å^−1^. Extraction of the scattered intensity I as a function of the scattering angle 2θ was obtained from the azimuthal angular integration [0, 2π] of the diffraction patterns with a home-made developed software.

Thermogravimetric analyses (TGA, TGA 4000 Instruments, Perkin Elmer (Waltham, MA, USA), 100–240 V/50–60 Hz) were performed on nanoparticle samples dried at 60 °C in an oven for two days. Approximately 20 mg of samples were automatically weighted and heated from 30 to 600 °C at a heating rate of 3 °C/min with an oxygen flow of 20 mL/min. The associated efficiency of CD-MO on RT MIL100 was calculated based on the dried weight and the mass of residues in CD-MO@RT MIL100.

Attenuated Total Reflectance (ATR, PerkinElmer, FT-IR Spectrometer, UATR Two) technology was utilized to analyze the structure and compositional information of nanoparticles as dried powders.

#### 3.2.6. Characterization of CD-MO

Proton nuclear magnetic resonance spectroscopy (^1^H NMR, Bruker Avance 360 MHz) was employed to analyze the composition of CD-MO by dissolving samples in deuterated water (D_2_O). The ^1^H NMR analysis method of CD-MO was the same as our reported CD-citrate oligomers [27]. The ratio of malic acid and CD was calculated by integrating the peaks of malic acid (4 protons of methylene group) and CD units (7 anomeric protons). The CD wt % content can be further calculated based on the molecular weights of the β-CD (1135 g/moL) and the malic (134 g/moL) moieties.

The number and weight average molar masses (Mn and Mw respectively) of CD-MO were analyzed with Size-exclusion chromatography (SEC, guard columns OPAK SB-G and two columns in series OHPAK SB 804 et 806 HQ), coupled online with multi-angle light-scattering (MALS, Dawn Heleos II Wyatt Technology, Santa Barbara, CA, USA) and differential refractive index (RI, RID 10 A Shimadzu, Kyoto, Japan) detectors (SEC/MALS/RI,). A total of 100 µL of 0.45 µm filtered CD-MO solutions (10 g·L^−1^) in LiNO_3_ 0.1M were injected for analysis. 0.1 µm filtered LiNO_3_ 0.1 M, has been used as eluent at 0.5 mL·min^−1^. A dn/dc value of 0.14 mL·g^−1^ has been used for CD-MO [45].

Data were determined thanks to the Astra^®^ 6.1.7 software from Wyatt Technology using Zimm order 1 between 44.8° and 140°.

### 3.3. Drug Encapsulation and Release

#### 3.3.1. AMP Encapsulation and Payload Efficiency Test

A suspension of 1 mg/mL RT MIL100 was first prepared. A total of 1 mL of this suspension was added into 1 mL AMP aqueous solution (0.2 mg/mL) under stirring at 500 rpm for 24 h at room temperature. The weight ratio between RT MIL100 and AMP was 5:1. Samples were collected by centrifugation (10,000× *g*, 10 min), and the supernatant was recovered for further high performance liquid chromatography (HPLC) investigations.

Likewise, AMP was loaded within CD-MO@RT MIL100. A 1 mL suspension of CD-MO@RT MIL100 (1 mg/mL) was added dropwise into 1 mL 0.2 mg/mL AMP aqueous solution, under stirring at 500 rpm for 24 h at room temperature. The weight ratio between CD-MO@RT MIL100 and AMP was 5:1. Samples were collected by centrifugation (10,000× *g*, 10 min), and the supernatant was recovered for HPLC quantification.

The drug encapsulation efficiency (EE) was calculated following Equation (1):

(1) $$\mathrm{EE}\;\left(\%\right)=\frac{\mathrm{encapsulated}\;\mathrm{drug}\;\left(\mathrm{mg}\right)}{\mathrm{initial}\;\mathrm{drug}\;\left(\mathrm{mg}\right)}\times 100;$$

The drug payload efficiency was determined based on Equation (2):

(2) $$\mathrm{Payload}\;\left(\%\right)=\frac{\mathrm{encapsulated}\;\mathrm{drug}\;\left(\mathrm{mg}\right)\;}{\mathrm{RT}\;\mathrm{MIL}100\;\left(\mathrm{mg}\right)}\times 100$$

#### 3.3.2. AMP Release and RT MIL100 Degradation

After the preparation of AMP@RT MIL100 and CD-MO@RT MIL100-AMP, supernatants were collected for EE and payload determination. The pellets were re-dispersed in 1 mM PBS for drug release and degradation studies.

To study AMP release from RT MIL100, AMP@RT MIL100 suspensions were centrifuged at 10,000× *g* for 10 min, and the pellets were re-dispersed in 4 mL 1 mM PBS. The resulting suspensions contained 0.25 mg/mL AMP@RT MIL100. Aliquots of 400 μL were separately distributed into Eppendorf tubes and mildly rotated at 37 °C. At various times (0 h, 0.5 h, 1 h, 2 h, 4 h, 6 h, and 24 h), one sample was taken and centrifuged (10,000× *g*, 10 min), and the supernatant was evaluated by HPLC. The same strategy was used in the case of coated nanoparticles.

HPLC analysis was carried out with an Agilent system that includes a tunable UV absorbance detector. A C18 Silica column (4.6 × 250 mm, 5 mm; Phenomenex) was employed with an eluant flow of 0.5 mL/min at 25 °C. The mobile phase comprised 88% buffer (0.2 M TEAA) and 12% methanol. AMP was detected at 254 nm with an injection volume of 10 μL. Additionally, the degradation of RT MIL100 was studied in 1 mM PBS according to the release of trimesate.

### 3.4. Cell Toxicity Study

The toxicity of RT MIL100 and CD-MO was investigated using an MTT test. Firstly, macrophage J744 cells were seeded in 96-well plates with 10,000 cells/well. After incubating at 37 °C for 24 h, cells were treated with various concentrations of CD-MO or RT MIL100 coated or not with CD-MO. Nanoparticle suspensions and CD-MO solutions were prepared in sterile water. A total of 20 μL of nanoparticle suspensions were added into each well, to reach final concentrations of 0–0.4 mg/mL. After 4 h incubation, the DMEM media were discarded from each well and the cells were washed with 200 μL PBS, followed by the addition of 200 μL fresh DMEM. Then the cells were incubated overnight. An MTT solution was prepared by dissolving MTT powder in 1× DPBS, and the solution was filtered (0.22 μm). Then, MTT solution (20 μL, 5 mg/mL) was added into each well and incubated for 1 h. Finally, MTT media were removed, and 150 μL DMSO was added to dissolve formazan crystals. The absorbance was detected with a plate reader (GloMax, Discover Microplate Reader, Promega, Sunnyvale, CA, USA) at a wavelength of 460 nm.

## 4. Conclusions

RT MIL100 was successfully obtained using acetic acid as a modulator which effectively allowed for reduction of the size. This study highlighted the stability challenges arising when reducing the size of RT MIL 100. To circumvent this issue, novel CD-MO oligomers were synthesized to obtain core-shell CD-MO@RT MIL100 endowed with long-term stability and in which the model drug AMP was loaded with efficiencies close to 98 % reaching payloads of 19 wt %. In vitro cell studies showed the negligible toxicity of RT MIL100 and CD-MO. Further studies will focus on anticancer applications of drug-loaded RT MIL 100 nanoparticles.

## Figures and Tables

**Table 1:** Characteristics of the CD-MO oligomers as a function of the reaction time and temperature, as well as the molar ratio between the reactants, CD and malic acid.

Series	Temperature (°C)	Reaction Heating Time (Min)	M_n_ (g/mol)	M_w_ (g/mol)	Ð	Yield (%)	Molar Ratio CD: Malic
1	160	25	5200	11,700	2.2	24%	1:3
2	160	30	9200	26,900	2.9	27%	1:4.8
3	160	45	12,300	37,900	3.1	30%	1:4.5
4	160	60	16,700	54,400	3.3	37%	1:3.8
5	160	90	23,000	90,600	3.9	38%	1:3.6
6	170	20	8000	32,300	4.0	30%	1.4.4
7	170	25	9600	35,000	3.6	32%	1:4.3
8	170	30	9200	33,100	3.6	31%	1:3.7
9	170	45	11,800	48,000	4.1	33%	1:3.6
10	180	20	15,600	55,300	3.5	30%	1:4
11	180	25	16,600	65,700	3.9	35%	1:3
12	180	30	15,600	54,800	3.5	24%	1:5.7

## Data Availability

Data are available upon request.

## Supplementary Materials

The supporting information can be downloaded at: https://www.mdpi.com/article/10.3390/ijms24021757/s1.
