# Supplementary material for: Acetic Acid-Modulated Room Temperature Synthesis of MIL-100 (Fe) Nanoparticles for Drug Delivery Applications
Source: Int J Mol Sci. 2023 Jan 16;24(2):1757. doi: 10.3390/ijms24021757 (PMC9866736; doi:10.3390/ijms24021757)
Supplement: Supplementary file 1 [file ijms-24-01757-s001.zip › ijms-2142167-SI.pdf]

SUPPORTING INFORMATION

Acetic acid-modulated room temperature synthesis of MIL100 (Fe) nanoparticles for drug delivery applications

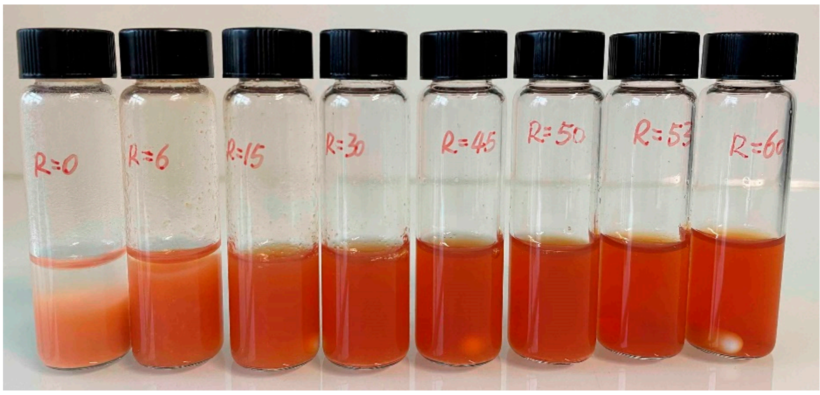

Figure S1. Suspensions of modulated RT MIL100 (R=0-60) after storage at room temperature for two days.

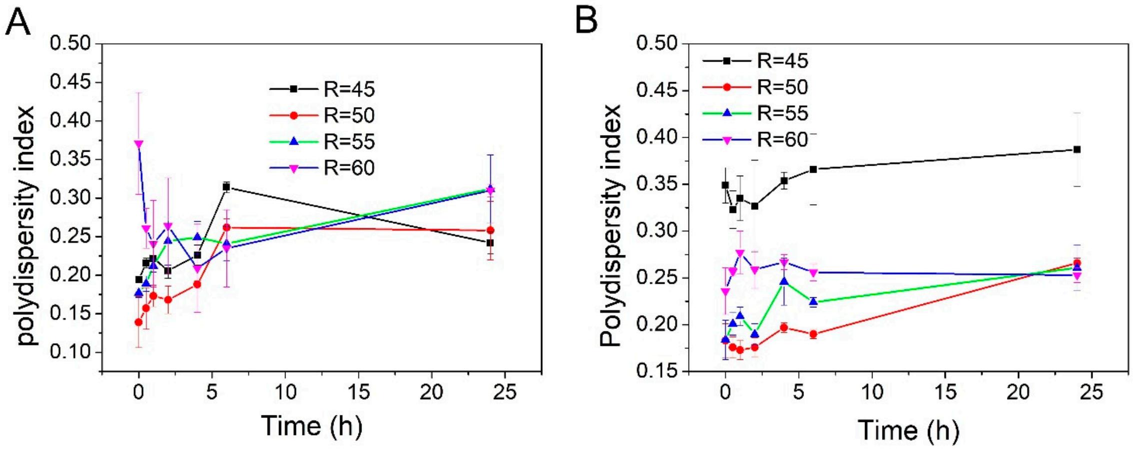

Figure S2. Polydispersity index of RT MIL100 with different molar ratios of the modulator and trimesic acid (R=45, 50, 55, 60) at 500 rpm (A) and 300 rpm (B).

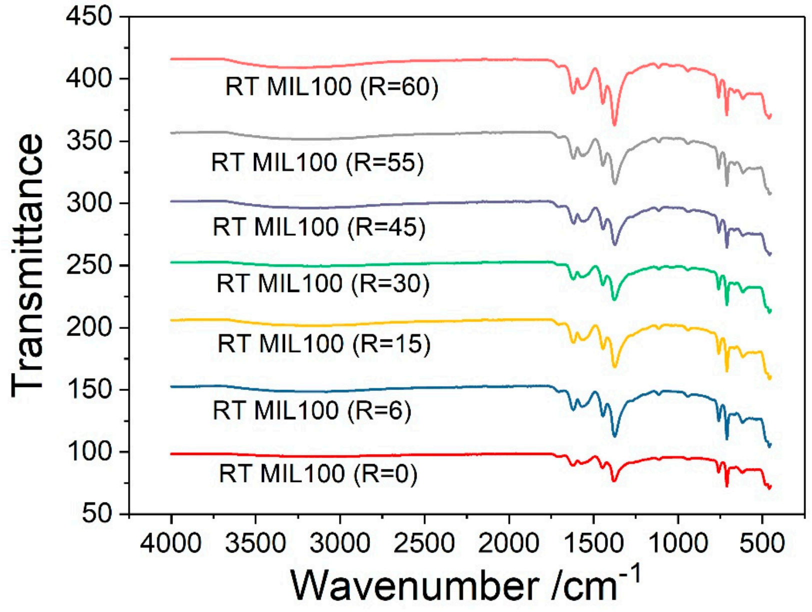

Figure S3. ATR infrared spectrums of RT MIL100 synthesized with different concentrations of modulators (R=0-60).

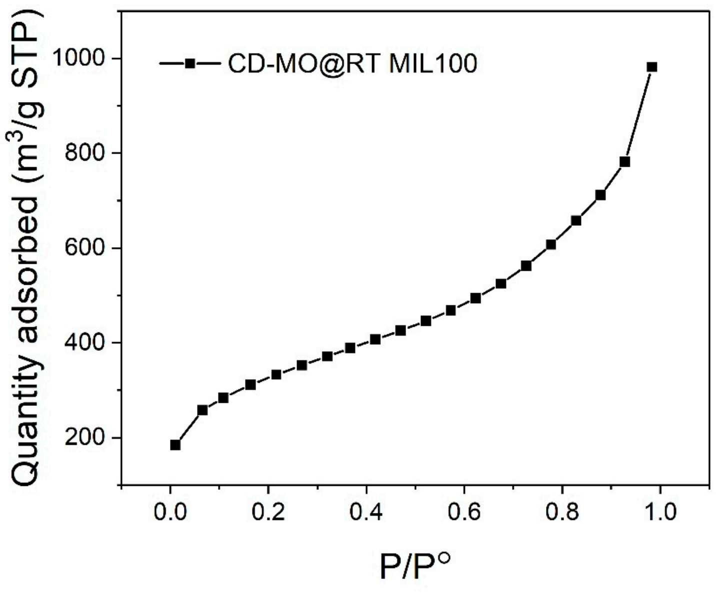

Figure S4. Porosimetry characterization of CD-MO@MIL100 (R=53) at 77.3 K;

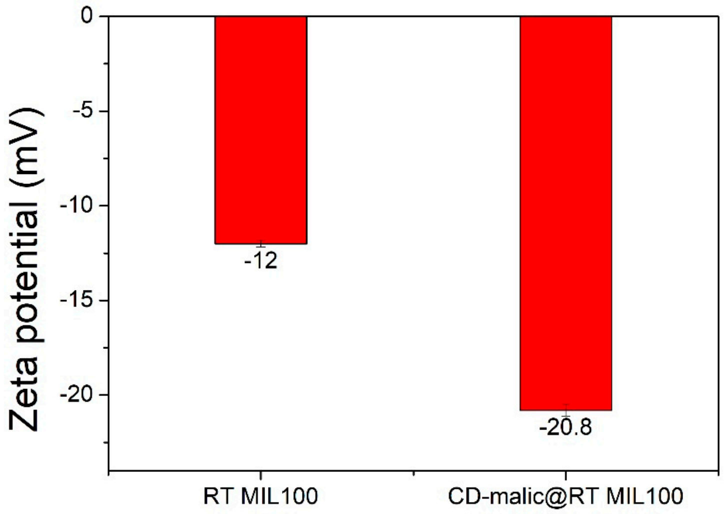

Figure S5. Zeta potentials of RT MIL100 and CD-MO@RT MIL100 in aqueous solution.

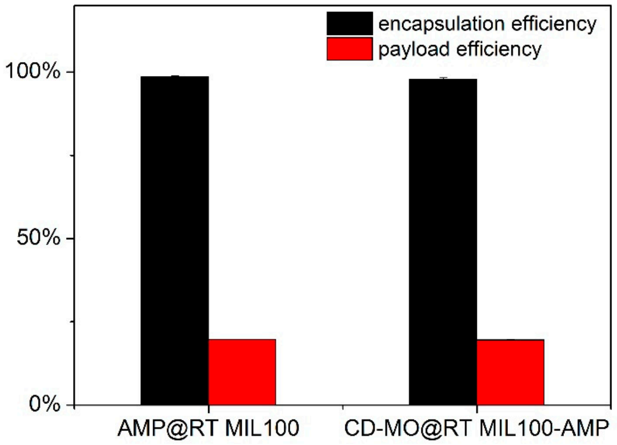

Figure S6. Encapsulation efficiency and payload efficiency of AMP@RT MIL100 and CD-MO@RT MIL100-AMP.
